# Traffic Sign Detection Based on SSD Combined with Receptive Field Module and Path Aggregation Network

**DOI:** 10.1155/2022/4285436

**Published:** 2022-05-30

**Authors:** Jianjun Wu, Shaowen Liao

**Affiliations:** College of Information Technology and Communication, Hexi University, Zhangye 734000, China

## Abstract

The traditional traffic sign detection algorithm can not deal with the application scenarios such as intelligent transportation system or advanced assisted driving environment, and it is difficult to meet the application requirements in detection accuracy and efficiency. Focusing on the above problems, this paper proposes a traffic sign detection algorithm based on Single Shot Multibox Detector (SSD) combined with Receptive Field Module (RFM) and Path Aggregation Network (PAN). The proposed algorithm is abbreviated to SSD-RP. The SSD-RP uses the RFM to improve the receptive field and semantics of the predicted feature maps, thus improving the detection performance of small traffic signs. At the same time, the path aggregation network is introduced to integrate multiscale features, which makes the abstract semantic information and rich detailed information shared among multiscale feature maps, enhances the discrimination ability of feature system, and improves the location and classification accuracy of traffic signs. Following that, the spatial pyramid pooling module is used to pool the shallow features and integrate them into the bottom-up information transmission path of the path aggregation network, thus continuing to supplement the fine-grained features for the feature system and further improve the detection performance. The experimental results on GTSDB and CCTSDB data sets show that SSD-RP has higher mean average precision (mAP) than traditional SSD algorithm and can better detect small traffic signs, which means that SSD-RP has higher detection precision. In addition, the experimental results also show that, compared with the common object detection algorithms such as Faster R-CNN, RetinaNet, and YOLOv3, the SSD-RP achieves a better balance between detection time and detection precision.

## 1. Introduction

With the rapid development of economy and people's demand for fast and convenient travel, the number of vehicles keeps increasing, and the incidence of road traffic jams and traffic accidents is increasing. Following that, the intelligent traffic system (ITS) has appeared [1]. As an important part of ITS, traffic sign detection provides road traffic information, so that drivers can make more reasonable driving decisions based on it, and it is also conducive to ensuring driving safety and reducing the incidence of traffic accidents. However, the actual road environment is changeable and complex, which caused some great difficulties to the traffic sign detection. How to ensure the detection speed and accuracy of traffic sign detection algorithm has always been one of the hotspots in the ITS.

In the early period of traffic sign detection, the shape and color are generally used to traffic sign detection. However, shape and color are easy to be interfered by the external information, so it is difficult to obtain the ideal detection performance in the actual scenarios. Some more abstract features are used to solve the detection problems of traffic sign along with emergence and continuous development of machine learning. Compared with previous detection algorithms, the detection accuracy has been improved. In recent years, driven by deep learning, some new algorithms gradually surpassed traditional algorithms in terms of traffic sign detection, as a special case of object detection has also been deeply influenced.

Traditional detection algorithm based on color: a traffic sign usually contains specific and own unique color and shape information. For example, in China's traffic signs, blue represents indication, yellow represents danger, and red represents prohibition. The most common shapes of traffic signs are usually square, triangle, and circle. Therefore, in the early stage, traffic sign detection is generally based on color and shape information. Wang et al. [2] developed a system that makes good use of color information, which can operate accurately at high speed and under various weather conditions and can identify all traffic signs from the online road sign database. Wang et al. [3] put forward a method for red bitmap based on edge information. The recall rate of prohibition signs and danger signs on GTSDB traffic sign data set reaches 99% and 97%, respectively, and the accuracy remains above 99%.

Traditional detection algorithm based on shape: because the surface color of traffic signs may change, but the shape remains unchanged, this can improve the detection performance. Chen and Lu [4] proposed a traffic sign detection technology by studying the application of support vector regression (SVR) in discriminant detector learning. This method establishes a significance model, and good results have been achieved. Aiming at the problems of various weather conditions often encountered in the actual traffic video acquisition, Chen et al. [5] put forward a distinctive method for shape matching, which has strong robustness. In Tang et al. [6], aiming at the serious problem of missing detection of traffic signs due to color distortion, shape distortion, and scale change, a multifeature cooperation method is proposed. This method calculates the color enhancement image from the traffic scene image, uses the multithreshold segmentation method and the chain code expression of the closed contour curvature histogram, integrates the color features and the normalized shape features to form the feature vector of the region, and obtains the detection results.

Traditional detection algorithm based on machine learning: by designing more representative features and using effective classifiers, machine learning algorithm has achieved better results in traffic sign detection than image processing. Ardianto et al. [7] used HOG and SVM to realize the traffic sign detection system, and the accuracy reaches 90%. Liu et al. [8] mainly focused on the recognition of small and medium-sized object in large scenes and proposed a detection framework based on attention context area (AC-RDF), which eliminated the gap between detection and classification. For Liang et al. [9] to solve this problem of surrounding environment interference in the road image in front of the vehicle collected by the vehicle camera, an adaptive recognition method of road traffic signs based on double edge Hough detection of lane line and primary and secondary weighting method is proposed. The algorithm performs well in the case of multilane coexistence and environmental interference, without human intervention, and has good adaptability.

Traffic sign detection algorithm based on deep learning: with the development of related theories of deep learning, scholars have applied the deep learning algorithm with excellent performance to the traffic sign detection task and achieved good detection results. In 2015, Yang et al. [10] published the CTSD, the first data set of traffic signs in China, and proposed a fast traffic sign detection method, which used CNN to carry out detailed classification of traffic signs. The performance of GTSDB and CTSD data sets was similar to that of the most advanced detection methods at the same time with a faster detection rate. In order to solve the limitation of predefined traffic signs, Changzhen et al. [11] used the regional proposal network and CNN model to detect Chinese traffic signs in 2016. The model can still achieve more than 99% accuracy under the condition of real-time detection. In the same year, Tsinghua University and Tencent Lab jointly launched TT100K dataset [12] and proposed an 8-layer CNN for this dataset, which achieved 91% recall rate and 88% accuracy rate. In 2018, Natarajan et al. [13] proposed a low-complexity detection method using the weighted combination of multiple groups of parallel CNN networks, which achieved 99.59% accuracy on the GTSRB data set and could identify traffic sign images within 10 ms.

Yawar et al. [14] proposed the CTF method to gather semantic information in the network to improve the antagonism of D-patch, strengthen the feature extraction ability, and make the network more robust to the occlusion problem. The accuracy rate of GTSDB data set can reach 100%. The performance on the Korean traffic sign data set was 2.2% higher than before the improvement. Borui et al. [15] proposed IoU-Net network to obtain more accurate location detection boundary frames. The network was used to train intersection-over-Union (IoU) branches and extract positioning confidence of each boundary frame, thus improving positioning accuracy. It is also proposed that PrRol-Pooling solves the problem of location information loss to a certain extent during the whole operation of Rol Pooling in Faster RCNN. In 2019, Li and Zengfu [16] used CNN, an efficient and powerful classifier with an asymmetric kernel, as a classifier to improve the classification effect under the condition of reducing model parameters and computation. The accuracy of this algorithm on GTSRB data set was 99.66%. Since IoU value is always zero when the prediction frame and the real frame do not overlap and cannot represent the distance between them, Rezatofighi et al. [17] proposed Generalized Intersection-over-Union (GIoU) loss function, which can improve the accuracy of 2%–14%. Zhang et al. [18] proposed an traffic sign detection related methods “DetReco” based on yolov3 and CRNN, which can detect objects and texts in various scenes and detect and recognize affine transformed or occluded texts. Tabernik and Skocaj [19] proposed a detection model based on Mask R-CNN and data enhancement technology based on set and appearance distortion in 2020, which solved the problem of large-scale and multitype traffic sign detection. Ayachi et al. [20] established a traffic sign detection data set with 10500 images including 73 traffic sign categories by using Chinese roads photographed under real environmental conditions. The improved deep learning algorithm achieves an average accuracy of 84.22% on established data set. Liang et al. [21] proposed a sparse R-CNN that integrates coordinate attention block with ResNeSt, and better performance is obtained. Ahmed [22] proposed a new network to enhance traffic sign areas in images of complex environments and used CURE-TSD sets to evaluate the effectiveness of the method, achieving 91.1% accuracy and 70.71% recall rate.

At present, most object detection algorithms adopt multiscale prediction strategy. Shallow features predict small objects, and deep features predict medium scale and large objects. Although the shallow feature retains rich details, its receptive field is small and is semantically weak, so it cannot provide strong discrimination ability when detecting small objects, and it is easy to cause the false detection of small objects. Although deep features have enough receptive fields, details are gradually lost in the downsampling process, which is easy to cause small objects to miss detection. This is also one of the reasons for the poor performance of current mainstream detection algorithms in small object detection task. For example, in vehicle-mounted video, traffic signs mostly appear in the form of small objects, so the direct application of the current detection algorithm cannot achieve better detection results. In addition, abstract semantic information and rich detail information cannot be shared among multiscale feature maps, and the detection features cannot be displayed as much as possible, which is not conducive to improving the detection performance.

Based on the above reasons, this paper studies traffic sign detection based on traditional object detection algorithm SSD. Traffic sign detection algorithm based on SSD combined with Receptive Field Module (RFM) and PAN is abbreviated. The proposed algorithm is abbreviated to SSD-RP. SSD-RP Receptive Field Module (RFM) is used to improve the Receptive Field and semantics of prediction feature maps, so as to improve the effect of small traffic sign detection. By introducing Path Aggregation Network (PAN) to integrate multiscale features, abstract semantic information and rich detailed information can be shared among multiscale feature maps, and the expressiveness of feature system can be enhanced. After that, shallow features are pooled by Spatial Pyramid Pooling (SPP) module and integrated into the bottom-up information transmission path of PAN, further supplementing the feature system with fine-grained features. Experiments on GTSDB and CCTSDB datasets verify the validity of SSD-RP.

## 2. Traditional Single Shot Multibox Detector (SSD) Algorithm

SSD is an end-to-end first-order object detection algorithm. On the basis of retaining the good classification performance of VGG16, using the natural pyramid structure of the CNN, an additional feature layer is constructed to make it have higher detection accuracy, which can be suitable for task scenarios with high detection accuracy. The following briefly describes the network structure, anchor box scheme, and loss function of the SSD object detection algorithm.

### 2.1. Experimental Environment Setting and Evaluation Index

**Figure 1:**
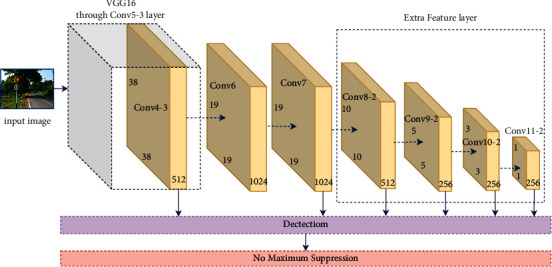
Network topology diagram of SSD algorithm.

As shown in Figure 1, the network structure of the SSD algorithm consists of backbone network, regression, and classification subnetworks.

The dotted arrow in the Figure 1 indicates the omitted convolution layer. The backbone network uses an improved VGG16 network. Compared with VGG16, SSD converts the fully connected layers FC6 and FC7 of VGG16 into 3 × 3 convolutional layers Conv6 and 1 × 1 convolutional layers Conv7, respectively, where the Conv6 layer is an Atrous Convolution with an dilated rate of 6 and a convolution kernel of 3 × 3, and the Conv7 layer is a point-by-point convolution with a convolution kernel of 1 × 1. All dropout layers and FC8 layers are removed, and eight additional convolution layers such as Conv8_1 and Conv8_2 are added to obtain feature maps of more scales. Among them, Conv8_1 is a 1 × 1 point-by-point convolution, and Conv8_2 is a downsampling convolution with a stride of 2 and a convolution kernel of 3 × 3. The pooling settings of the Pool5 layer are changed, the pooling window is changed from 2 to 3, and the step size is changed from 2 to 1. The feature map passes through this pooling layer, and the input resolution is preserved. The Atrous algorithm was added to obtain a denser score map with different receptive fields and resolutions.

The regression and classification subnetworks are used to generate the position offset of the predicted frame and the confidence level of the object in the frame. Both the regression network and the classification network are composed of standard convolutions with a convolution kernel of 3 × 3, but the dimension of the output of the regression subnetwork is *n* × 4, and the dimension of the classification subnetwork output is *n* × *m*, where *n* represents anchor boxes number generated by an anchor point, and *m* represents the number of categories prediction network.

### 2.2. Anchor Box Scheme

The SSD algorithm uses multiscale feature maps for prediction, making it possible to predict on different feature maps to improve detection accuracy, so they are also assigned anchor boxes of different scales, so that each feature map focuses on a specific scale object that is detected. The calculation of the anchor box reference size assigned to the predicted feature map is shown in the following equation:

(1) $$\begin{array}{c} s_{k}=s_{\text{min}}+\frac{s_{\text{max}}-s_{\text{min}}}{m-1}\left(k-1\right),\;k\in \left[1,\;m\right]. \end{array}$$

Among them, *s*_min_ and *s*_max_ are the benchmark sizes of the anchor boxes on the Conv4_3 and Conv11_2 feature maps, and the SSD algorithm uses six predicted feature maps, so *m* is 6. After the calculation of the above formula, the reference sizes of the anchor boxes allocated to the six predicted feature maps can be calculated in sequence. And for each anchor box, we set five different aspect ratios *ar*={0.3, 0.5, 1.0, 2.0, 3.0}, to fit objects of different shapes. For *ar*=1.0, a new benchmark size $s_{k}^{\prime }=\sqrt{s_{k}s_{k+1}}$ has been added. The width and height of the anchor frame can be calculated as follows:

(2) $$\begin{array}{c} \left\{\begin{array}{c} w_{k}^{a}=s_{k}\sqrt{a_{r},}\\ h_{k}^{a}=s_{k}\sqrt{a_{r}}. \end{array}\right) \end{array}$$

It is pointed out that the SSD algorithm only assigns anchor boxes with 4 aspect ratios on the Conv4_3, Conv10_2, and Conv11_2 feature maps.

### 2.3. Loss Function

The detection task is composed of two subtasks of localization and recognition. Therefore, when the SSD algorithm is used for object detection, the loss function is usually composed of the weighted sum of the localization loss and the confidence loss, as shown in the following equation:

(3) $$\begin{array}{c} \left(L\left(x,c,l,g\right)=\frac{1}{N}\left(L_{\text{conf}}\left(x,c\right)+\alpha L_{\text{loc}}\left(x,l,g\right)\right)\right), \end{array}$$

where *N* is positive samples number, and if *N* is 0, set the loss to 0. *α* is the weight factor, which is used to adjust the ratio between classification and localization losses, and the default is 1. Among them, the confidence loss uses the cross-entropy function as its classification loss function, and the function definition is shown in the following equation:

(4) $$\begin{array}{c} L_{\text{conf}}\left(x,c\right)=-\sum_{i\in Pos}^{N}x_{ij}^{p}\log\left({\hat{c}}_{i}^{p}\right)-\sum_{i\in Neg}\log\left({\hat{c}}_{i}^{0}\right), \end{array}$$

where *i* refers to the search box number, *j* refers to the real box number, *p* refers to the category number, and *p*=0 means background. The first half of the equation is the loss of the sample matching the anchor box with the real box, that is, the loss of classification as a certain category (excluding the background), and the second half is the loss of the anchor box where the sample does not match the real box; that is, the category is loss of the background. *x*_*ij*_^*p*^ indicates whether the predicted box *i* matches the real box *j* with respect to the category *p*, and it is 1 when it matches, and 0 when it does not match. Both positive samples and negative samples participate in the calculation of the classification loss function.

The confidence score ${\hat{c}}_{i}^{p}$ is generated by Softmax, and its definition is shown in the following equation:

(5) $$\begin{array}{c} {\hat{c}}_{i}^{p}=\frac{\exp\left(c_{i}^{p}\right)}{\sum_{p}\exp\left(c_{i}^{p}\right)}. \end{array}$$

The positioning loss function of the SSD algorithm is shown in the following equation:

(6) $$\begin{array}{c} L_{\text{loc}}\left(x,l,g\right)=\sum_{i\in Pos}^{N}\sum_{m\in \left\{cx,cy,w,h\right\}}x_{ij}^{k}{\text{smooth}}_{L1}\left(l_{i}^{m}-{\hat{g}}_{j}^{m}\right). \end{array}$$

Among them, the function smooth_*L*1_ is defined as shown in the following equation:

(7) $$\begin{array}{c} {\text{smooth}}_{L1}=\left\{\begin{array}{cc} 0.5x^{2}, & \left|x\right|<1,\\ \left|x\right|-0.5, & \left|x\right|\geq 1. \end{array}\right) \end{array}$$

Among them, *l*_*i*_^*m*^ represents the position change of the anchor box predicted by the network, and ${\hat{g}}_{j}^{m}$ represents the position change of the real box relative to the matched anchor box. It is seen that only positive samples participate in the calculation of the positioning loss function.

## 3. Traffic Sign Detection Based on SSD Combined with RFM and PAN

### 3.1. Receptive Field Module (RFM)

**Figure 2:**
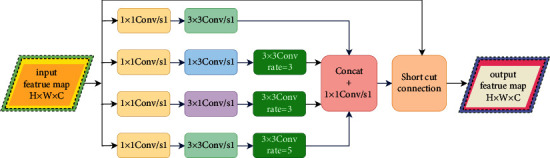
Topological structure of receptive field module (RFM).

The receptive field is the area where the feature points in the feature map are mapped back to the input image, and it reflects the amount of original image information required to calculate the current feature. The improvement of the receptive field can introduce context information, and through the comparison of the previous and background information, the model's ability to distinguish the object can be enhanced. Based on this, this paper designs a new Receptive Field Module (RFM). The topology of RFM is shown in Figure 2. The structural design of this module is inspired by the Inception module in GoogLeNet [23], which adopts a parallel branch structure and combines Atrous Convolution to make each branch have a different receptive field. At the end of the module, the outputs of different branches are spliced in the channel dimension, and the spliced feature map can express at multiple scales. Finally, the residual connection operation is used to enhance the information flow of the module.

As can be seen from Figure 2, the RFM topology has four branches: the top branch is named branch I, and the rest are analogous. In the four branches, the input feature maps are first subjected to a 1 x 1 point-by-point convolution, reducing channels number of the feature map to 1/4 of the input. Branch I is equipped with 3 x 3 standard convolutions. Branch II is equipped with a 1 x 3 standard convolution and an Atrous Convolution with a dilated rate of 3, the convolution kernel size is 3 x 3, and the padding is 3. Branch III is equipped with a 3 x 1 standard convolution and an Atrous Convolution with a dilated rate of 3, the convolution kernel size is 3 x 3, and the padding is 3. Branch IV is equipped with a 3 x 3 standard convolution and an Atrous Convolution with a dilated rate of 5, the convolution kernel size is 3 x 3, and the padding is 5. At the end of the RFM module, the outputs of the four branches are concatenated in the channel dimension, and then 1 x 1 point-wise convolution is used for interchannel information fusion. Finally, the input of the RFM module is shortcut connected with the fused features to enhance the information flow of the module. Each branch in RFM has a different receptive field because it uses different sizes convolution kernels and Atrous Convolutions with different dilated rates.

### 3.2. Path Aggregation Network (PAN)

Generally speaking, shallow features are closer to the input image and retain more detailed information. The deep features are mapped by multiple convolutional layers and have more semantic information. As a result, the features of different layers can be fused to improve their expressive ability. Feature Pyramid Network (FPN) is one of the most commonly used feature fusion methods at present. The network uses horizontal connections and top-down connections to pass down deep features with strong semantics and fuse them with shallow features. This fusion method enables strong semantic information to be shared among feature maps of different scales, which can effectively improve the semantics and receptive fields of shallow features. However, the deep features lack detailed information, and simple upsampling operations cannot recover the details well. In view of the above shortcomings, in order to better perform feature fusion, this paper introduces path aggregation network (PAN), which supplements detailed information for deep features by propagating the response of shallow features.

**Figure 3:**
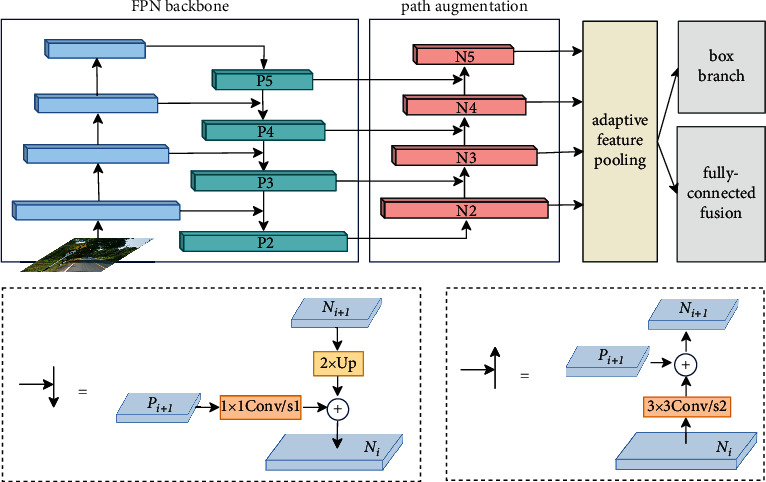
Topological structure of path aggregation network (PAN).

PAN adds a bottom-up information transmission path on the FPN, and its topology is shown in Figure 3. In the top-down information transfer path, the predictive feature map generated by the backbone network is first selected as the input of the FPN. Then, point-by-point convolution is used to change the channel dimension of the input feature map, so that the feature maps of different scales are consistent in the channel dimension for feature fusion. Then, the deep feature map doubles its resolution through an upsampling operation to make it consistent with the shallow feature map resolution, which can also be implemented using transposed convolutions. Finally, the feature fusion is realized by adding the deep feature map with the same number of channels and the same resolution to the shallow feature map element by element. In the bottom-up information transfer path, the output of the FPN serves as the input of the PAN. The resolution of the input feature map is first halved by downsampling convolution with stride 2 to make it consistent with the resolution of the deep feature map; after that, the shallow and deep features are fused by element-wise addition. The fused features have both the detail information of the shallow features and the semantic information of the deep features, which can improve the positioning accuracy and classification accuracy.

### 3.3. Spatial Pyramid Pooling (SPP)

**Figure 4:**
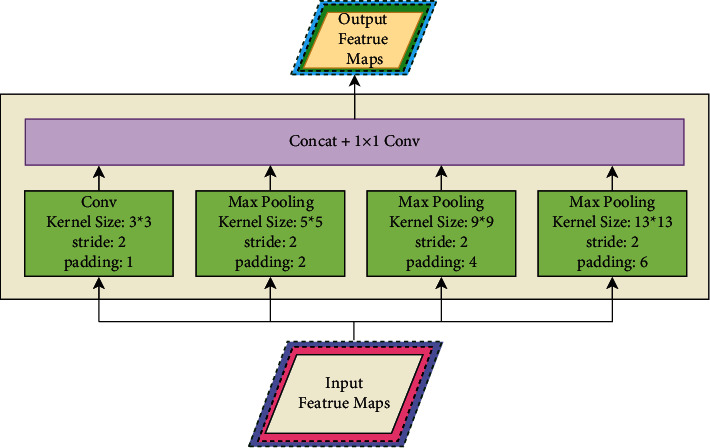
Topological structure of spatial pyramid pooling (SPP).

Inspired by the upward propagation of shallow feature responses from the PAN structure, it is hoped to obtain more fine-grained features to participate in the bottom-up information transfer to further enhance the details of the entire feature system. Therefore, this paper uses spatial pyramid pooling (SPP) for the shallow feature map. Different from the SPP proposed by He et al. [24], the topology of the SPP is shown in Figure 4. The main purpose is to downsample the resolution of the feature map to facilitate feature fusion with the deep feature map.

As shown in Figure 4, the SPP designed in this paper also adopts the structure design of parallel branches. Except branch I that uses standard convolution with stride 2, the remaining three branches use the maximum pooling operation with stride 2. The convolution kernel size is set to 3 x 3, and the pooling window is set to 5, 9, and 13, respectively. After the structure is designed in this way, the four branches have receptive field sizes of 3, 5, 9, and 13, respectively. While downsampling feature maps, multiscale spatial information can also be obtained. At the end of the structure, the outputs of the four branches are spliced together in the channel dimension, followed by point-by-point convolution to reduce the dimension and perform information fusion between channels, and the fused features are more robust.

### 3.4. The General Network Structure of SSD-RP

**Figure 5:**
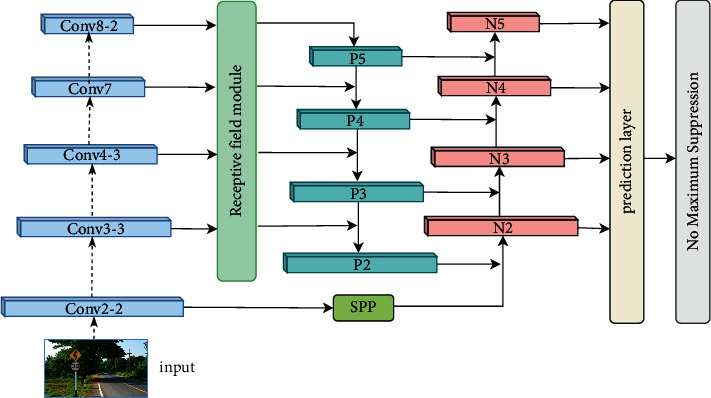
The general network structure of the SSD-RP algorithm.

For the above improvement strategy, this paper proposes a traffic sign detection algorithm based on SSD combined with RFM and PAN (abbreviated as SSD-RP). The general structure of the algorithm is shown in Figure 5.

The SSD-RP algorithm uses four predictive feature maps to detect traffic signs, so it only needs to keep the four convolutional layers in the original backbone network. The dotted arrow in the Figure 5 indicates the omitted convolution layer. SSD-RP first inputs the first-layer prediction feature map Conv3_3 into RFM. The module adopts the structure design of parallel branches, and each branch is equipped with convolution operations of different scales and different dilated rates, which can perform multiscale feature learning and effectively increase receptive field and semantics of input features. After that, the outputs of the RFM and the other three selected predictive feature maps are input into the PAN, which contains two information transfer paths, top-down and bottom-up. In the former path, deep features are passed down to provide semantic guidance for shallow features. In the latter path, shallow features are passed up to supplement detailed information for deep features. The fused features have both abstract semantic information and rich detailed information, which is beneficial to improve the positioning accuracy and classification accuracy. At the same time, the SPP module is used to pool the Conv2_2 feature map. The pooled output has the same resolution as the first-layer feature map in the PAN, which is easy to integrate into the bottom-up information transmission path of the PAN and further supplement the feature system with fine-grained feature. Finally, the output of pan is input into the prediction layer to generate the prediction box, and then the NMS operation is followed to filter out the prediction box with local regional redundancy to obtain the final detection result.

## 4. Traffic Sign Detection Experiments and Analysis of Results

### 4.1. Experimental Data

The Institute for Neural Computation in Germany has published a real-life Germany Traffic Sign Detection Benchmark GTSDB. This dataset collects road scenes from more than 60 cities in Germany, and the environment involves different moments, weather conditions, and seasons. The GTSDB dataset contains 900 images of street scenes in their natural environment. Among them, 600 images will be used as training set data, and the rest are divided into the test set. The resolution of each image is 1360 x 800 pixels, and 0 to 6 traffic signs are contained in each image. GTSDB dataset divides traffic signs into three main categories based on color and shape, namely, circular indication signs on a blue background, triangular danger signs with a red border on a white background, and circular prohibition signs with a red border on a white background.

CSUST Chinese Traffic Sign Detection Benchmark (CCTSDB) [25] was completed by Zhang Jianming's team from Hunan Key Laboratory of Intelligent Processing of Integrated Transportation Big Data of Changsha University of Science and Technology. The dataset has more than 15,000 images of street scenes in their natural environment, with resolutions between 600 × 900 and 1024 x 768 pixels. The size distribution of the traffic signs in the image ranges from 20 x 20 to 573 x 557 pixels. The signs in this dataset are currently divided into three categories, prohibitory sign, warning sign, and mandatory sign, and a subcategory of the standard dataset is still being developed.

### 4.2. Evaluation Indicators for Traffic Sign Detection

Traffic signs often contain several categories. For a single category A, the combination of the algorithm's prediction result and the sample true result can be classified into four categories; that is, if the algorithm's prediction result is category A, and the true result is also category A, the prediction is said to be a True Positive (TP) case. If the algorithm predicts a class A result, but the true result is not class A, the prediction is said to be a False Positive (FP). If the algorithm predicts that the result is not class A, and the true result is not class A, then the prediction is called True Negative (TN). If the algorithm predicts that the result is not class A, but the true result is class A, the prediction is called False Negative (FN). Recall, also referred to as Recall Ratio, represents the probability of a positive case being detected. Precision, also known as accuracy, represents the probability of the positive cases being detected by the algorithm, and the true category is also a positive case. The equations for the two are equations (8) and (9), respectively.

(8) $$\begin{array}{c} \text{Recall}=\frac{\text{TP}}{\text{TP}+\text{FN}}, \end{array}$$

(9) $$\begin{array}{c} \text{Precision}=\frac{\text{TP}}{\text{TP}+\text{FP}}. \end{array}$$

In traffic sign detection tasks, confidence thresholds can directly affect detection results, as well as recall and accuracy. In general, setting a high confidence threshold will result in a low recall and a high accuracy. Conversely, setting a lower confidence threshold will result in a higher recall and lower accuracy. Different detection scenarios have different preferences for recall and accuracy, and to comprehensively evaluate the performance of detection algorithms, mean Average Precision (mAP) is often used as a measure of detection precision. The mAP is calculated by setting different confidence thresholds and first calculating the recall and accuracy at different confidence thresholds. The PR curve is then plotted with accuracy as the vertical coordinate and recall as the horizontal coordinate. The area under the curve is the AP value for a single category, and the AP value is not affected by the confidence threshold. For multiple categories, the AP values for all categories are averaged to get mAP. The higher the mAP in object detection is, the more the objects can be detected correctly.

Traffic sign detection time is the time, in ms, taken by the algorithm to run the detection process. Detection time is an important metric for evaluating the speed of an algorithm's detection, with shorter detection times meaning faster detection. Detection speed can also be measured in terms of the number of frames per second (FPS) that can be processed. It is worth pointing out that the detection time needs to be compared on the same hardware device in order to be meaningful.

### 4.3. Experimental Environment for Object Detection

In this paper, traffic sign detection was performed on Ubuntu 18.02 and PyTorch 1.6.0. Several sets of comparison experiments were conducted, and the same training configuration was used for the models in the experiments in order to fairly compare the effectiveness of the methods proposed in this paper. The input image resolution is uniformly 300 × 300, and the training batch size is 8. The network is trained using a random gradient descent optimizer with an initial learning rate of 2*e*-4, a momentum factor of 0.93, and a weight decay factor of 1*e*-5. The backbone network is loaded with parameters using pretraining.

### 4.4. RFM Ablation Experiments

The RFM designed in this paper is mainly used to reinforce predictive features, so there are two available ways of using it. The first way is to embed it in the backbone network. The second is to put it in the branch structure. As four predictive features are used in this paper, there are various options for the number of RFMs to be used.  Table 1 demonstrates the impact of the RFM on object detection performance using different numbers of modules for the two modes of use.

In Table 1, “RFM-Embed” indicates the use of RFM embedded in the backbone network, with a mAP value of 91.2% and a detection time of 16 milliseconds. “RFM-Bypass” indicates the use of the RFM in a bypass structure with a mAP value of 91.8% and a detection time of 15 milliseconds. It indicates that the detection precision of the “RFM-Embed” is slightly lower than that of the “RFM-Bypass” method, probably because the “RFM-Embed” changes the backbone network structure, which makes the loss value of the algorithm larger in the early stage of training, and the convergence of the algorithm lower in the same number of iterations. There is also a small difference in detection time between the two methods, with the “RFM-Bypass” taking less time to detect, probably due to the parallel computing capabilities of the learning framework PyTorch, which facilitates the computation of the “RFM-Bypass.”

In considering the performance advantages in terms of detection precision and speed, the “RFM-Bypass” method is chosen for this paper. Once the usage has been determined, the number of RFMs is increased in turn, the layers Conv4_3, Conv7, Conv8_2, etc. are added gradually. In Table 1, the detection time for the “RFM-Bypass” method gradually increases by small amounts, but the corresponding mAP values do not change much. It shows that after the RFM has reinforced the first predictive feature map, there is no additional benefit gained by adding another RFM module cumulatively. This is because the subsequent predictive feature maps are compared to the small traffic signs. The receptive field and semantics are already sufficient, and overraising the receptive field would instead ignore detailed information. Therefore, based on the above analysis, it is possible to determine how, where, and how many RFMs should be used to enhance the receptive field and semantics of the predictive feature maps.

### 4.5. Ablation Experiments with the Feature Fusion Module

This paper compares the impact of three feature fusion methods, FPN, PAN, and PAN + SPP, on detection precision. The experimental results are shown in Table 2, where “Base” refers to the base network without feature fusion, which was used for the corresponding mAP value of 92.4%. The mAP value decreased by 0.9% instead after using the FPN feature fusion structure, because the deep features of the input image lacked details, a simple upsampling operation could not recover the detail well, and direct fusion with shallow features would make the feature representation more confusing. The PAN feature fusion structure corresponds to a mAP value of 92.8%. Since PAN is based on FPN with an additional bottom-up information transfer path that adds detail information to deep features, its mAP value is improved by 1.3% relative to FPN.

From Table 2 it can also be found that the combined PAN + SPP feature structure gets higher mAP values compared to PAN, indicating that fine-grained features have a greater role in small object detection tasks, which also implies that the combined PAN + SPP structure is more beneficial in feature fusion. The last row in Table 2 integrates the combined PAN + SPP feature structure and RFM to form exactly the SSD-RP algorithm proposed in this paper, and the mAP values obtained are the highest of all the cases.

### 4.6. Comparison of Detection Results between SSD-RP Algorithm and Traditional SSD Algorithm

**Figure 6:**
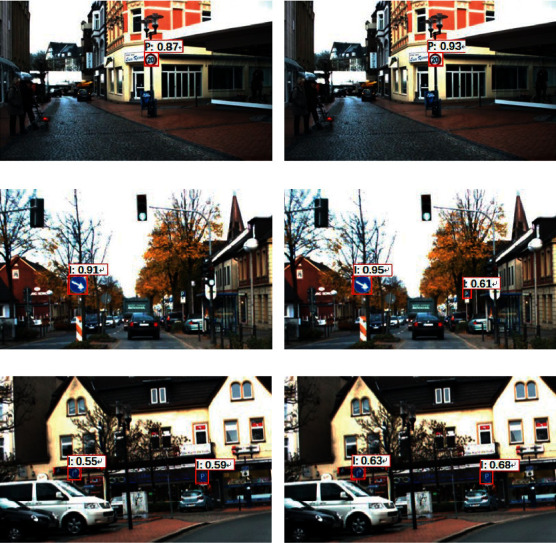
Visual comparison of SSD and SSD-RP detection results on the GTSDB dataset. (a) SSD detection result of No. 1 image. (b) SSD-RP detection result of No. 1 image. (c) SSD detection result of No. 2 image. (d) SSD-RP detection result of No. 2 image. (e) SSD detection result of No. 3 image. (f) SSD-RP detection result of No. 3 image.

The detection results of the traditional SSD algorithm are compared with those of the SSD-RP. A comparison of the visualization results on the data set GTSDB is shown in Figure 6. In the figure, the letters “D,” “P,” and “I” are abbreviations for the traffic sign categories, which stand for danger, prohibition, and indication traffic signs, respectively.

As seen in Figure 6, the SSD algorithm tends to miss the detection of small traffic signs because of the weak discrimination ability of the feature system. For example, SSD could not detect the small prohibited class traffic sign in Figure 6(c), while SSD-RP could detect this small sign in Figure 6(d). Also, by comparing the confidence in the figures, it is seen that, for the same traffic sign, the confidence of SSD-RP is generally higher than that of SSD, which means that the detection results of SSD-RP are more accurate than those of SSD.

The following is a quantitative comparison of the detection precision of the two algorithms. The indicators used are Average Precision (AP) and mean Average Precision (mAP), respectively, and the results are shown in Table 3. As seen in Table 3, SSD-RP obtained 95.4% mAP on the GTSDB dataset, which is a 2.2% improvement in mAP compared to SSD, indicating that SSD-RP has higher detection precision than SSD on the GTSDB dataset. In addition, Table 3 shows that both algorithms have higher detection precision for directional traffic signs, which may be due to the facts that directional traffic signs are more easily detected.

**Figure 7:**
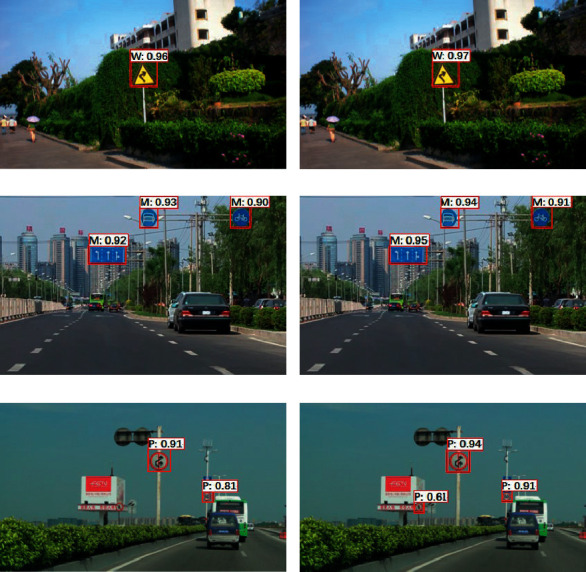
Comparison of SSD and SSD-RP detection results on the CCTSDB dataset. (a) SSD detection result of No. 1 image. (b) SSD-RP detection result of No. 1 image. (c) SSD detection result of No. 2 image. (d) SSD-RP detection result of No. 2 image. (e) SSD detection result of No. 3 image. (f) SSD-RP detection result of No. 3 image.

In order to verify the performance of the algorithm on a larger dataset, the detection results of the traditional SSD and the SSD-RP algorithm are compared on the dataset CCTSDB, and the visual detection results of the two algorithms are shown in Figure 7. In the figures, “M,” “P,” and “W” are abbreviations for the categories, which stand for prohibition sign, mandatory sign, and warning sign, respectively.

As can also be seen in Figure 7, the SSD tends to miss the detection of small traffic signs. For example, in Figure 7(e), SSD missed the detection of the small no-passing sign in the bottom right corner of the billboard, while, in Figure 7(f), SSD-RP could detect the small sign. Also by comparing the confidence values in the graphs, it can be seen that, for the same traffic sign, the SSD-RP generally has a higher confidence value than the SSD. For example, the right turn warning signs in Figures 7(e) and 7(b) have a confidence value of 0.94 for the SSD and 0.97 for the SSD-RP. The following is a quantitative comparison of the detection precision of the two algorithms on the CCTSDB dataset, the metrics used are still Average Precision (AP) and mean Average Precision (mAP), and the results of the comparison are shown in Table 4.

As seen in Table 4, SSD-RP obtained 95.9% mAP on the CCTSDB dataset, which is a 2.1% improvement in mAP compared to SSD, indicating that SSD-RP has a higher detection precision than SSD on the GTSDB dataset. Additionally, it can be seen from Table 4 that both algorithms have a higher detection precision for the prohibited class of traffic signs. Also comparing Table 4 with Table 3, the SSD-RP and SSD obtain a slightly higher mAP on the CCTSDB dataset than on the GTSDB dataset.

### 4.7. Comparison of SSD-RP with Other High Performance Detection Algorithms

In order to compare the performance of SSD-RP with other object detection algorithms more comprehensively, SSD-RP is now used simultaneously with traditional algorithms such as Faster R-CNN [26], RetinaNet [27], and YOLOv3 [28] for traffic sign detection in the GTSDB dataset, and the detection experimental environment remains the same as the above experiments.  Table 5 shows the performance comparison results of the above SSD-RP algorithms on the GTSDB dataset, and the table measures the performance of each algorithm in terms of detection time and mAP values respectively.

We see in Table 5 that Faster R-CNN used two stages to adjust and subclassify the anchor box to obtain the highest detection accuracy. RetinaNet uses focal loss to optimize the model parameters, which facilitates the network to focus on learning from difficult samples and performs well on small object detection tasks. In addition to the detection process and loss function, another reason for the superior performance of these two detection algorithms is the use of ResNet50 [29] as the deep backbone network. However, this approach generates a relatively large amount of computation during the detection process. The table shows that the single image detection times for these two algorithms are 98 ms and 90 ms, respectively, which indicates that the detection takes more time and is slower.

YOLOv3 in Table 5 is a typical one-stage detection algorithm, and because it uses a specially designed backbone network, DarkNet53, it has a fast feed forward speed, as seen in the table with a detection time of 35 ms, which is much less than the detection time taken by Faster R-CNN and RetinaNet. Also, YOLOv3 uses multiscale training, which allows the algorithm to perform well on small object detection tasks, with a mAP value of 93.8%. SSD-RP achieved a mAP value of 95.4% and a detection time of 26 ms for a single image, respectively, which is a good balance between detection time and detection precision compared to the previous algorithms. In addition, the SSD-RP algorithm improves detection accuracy by 2.2% compared to conventional SSDs but adds 7 ms to the detection time due to the additional computation introduced by the branched structure.

## 5. Conclusions

A novel traffic sign detection algorithm based on SSD combined with RFM and PAN is proposed. The SSD-RP uses RFM to improve the receptive field and semantics of predicted feature map and introduces PAN to fuse multi-scale features. Then, the SPP module is used to pool the shallow features and integrate them into the bottom-up information transmission path of the PAN. The ablation experimental results of RFM show that the use mode of “RFM bypass” has advantages in detection precision and detection speed. In practical application, the use mode and quantity of RFMs can be determined according to actual needs. The ablation experiment results of feature fusion module show that PAN + SPP combined structure has more advantages in feature fusion. If it is integrated with RFM, the highest mAP value can be obtained.

The comparison between SSD-RP and traditional SSD algorithm shows that SSD is easy to miss detecting small traffic signs because of its weak discrimination ability of feature system, while SSD-RP has stronger ability to detect small signs, and the detection confidence is generally higher than that of SSD. SSD-RP obtained 95.4% mAP on GTSDB dataset, increased by 2.2% compared with SSD mAP. SSD-RP obtained 95.9% mAP on CCTSDB dataset and increased by 2.1% compared with SSD mAP, indicating that SSD-RP has higher detection precision than SSD on both datasets. In addition, the experimental results also show that, compared with the common object detection algorithms such as Faster R-CNN, RetinaNet, and YOLOv3, SSD-RP can achieve a better balance between detection time and detection precision. At present, the calculation of SSD-RP algorithm is still relatively time-consuming, which has high requirements for the storage capacity and computing power of the device. The future work includes the performance improvement and lightweight design of SSD-RP algorithm.

## Figures and Tables

**Table 1:** Results of RFM ablation experiments.

	Con3_3	Conv4_3	Conv7	Conv8_2	Detection time (ms)	mAP (%)
RFM-embed	√				16	91.2

RFM-bypass	√				15	91.8
	√	√			17	91.7
	√	√	√		18	92.0
	√	√	√	√	20	92.5

**Table 2:** Ablation experiments for the feature fusion module.

Base	FPN	PAN	SPP	RFM	mAP (%)
√					92.4
	√				91.5
		√			92.8
		√	√		93.9
		√	√	√	95.4

**Table 3:** Comparison of detection precision between SSD and SSD-RP on the GTSDB dataset.

Detection algorithms	AP (%)			mAP (%)
	Indication sign-I	Danger signs-D	Prohibition signs-P	
SSD	97.7	92.3	89.5	93.2
SSD-RP	97.9	94.8	93.6	95.4

**Table 4:** Comparison of the detection precision of SSD and SSD-RP on the CCTSDB dataset.

Detection algorithms	AP (%)			mAP (%)
	Mandatory sign-M	Prohibitory sign-P	Warning sign-W	
SSD	96.9	90.8	93.8	93.8
SSD-RP	98.1	94.2	95.3	95.9

**Table 5:** Performance comparison of SSD-RP with other object detection algorithms on the GTSDB dataset.

Detection algorithms	Backbone networks in algorithms	Detection time (ms)	mAP (%)
Faster R-CNN	ResNet50	98	97.9
RetinaNet	ResNet50	90	96.7
YOLOv3	DarkNet53	35	93.8
SSD	VGG16	19	93.2
SSD-RP	VGG16	26	95.4

## Data Availability

The GTSDB and CCTSDB datasets used in this paper are open, which can be downloaded from the Internet.
